# Behavioral and Physiological Responses to Sensory and Cognitive Demands in an Immersive Virtual Reality Shopping Task in Unimpaired Young Adults: Within-Subjects Experimental Study

**DOI:** 10.2196/84893

**Published:** 2026-08-28

**Authors:** Salvatore Luca Cucinella, Job LA Mulder, Joost CF de Winter, Laura Marchal-Crespo

**Affiliations:** 1 Department of Cognitive Robotics Faculty of Mechanical Engineering Delft University of Technology Delft The Netherlands; 2 Department of Rehabilitation Medicine Erasmus Medical Center Rotterdam The Netherlands; 3 Department of Rehabilitation Medicine Rijndam Rehabilitation Centre Rotterdam The Netherlands

**Keywords:** virtual reality, head-mounted display, eye tracking, gaze behavior, head movement, heart rate, cognitive load, sensory demands, workload, shopping task, neurorehabilitation

## Abstract

**Background:**

Immersive virtual reality (IVR) is increasingly used for task training. Adjusting sensory and cognitive complexity to individual capacity is important for effective training, yet it remains unclear how varying visual, auditory, and cognitive demands influence behavioral responses, physiological activity, and task performance during goal-directed activities in IVR.

**Objective:**

This study aimed to examine how 2 levels (low and high) of visual, auditory, and mental demands affect self-reported mental demand and effort (MDE), task performance, heart rate, gaze behavior, and head movements during a virtual grocery shopping task.

**Methods:**

A within-subjects study was conducted with 22 unimpaired university students (11 women, 11 men; aged 23-27 years) recruited via convenience sampling at TU Delft, the Netherlands. Participants completed a grocery shopping task (collecting products from shelves during 122-second trials) under 7 conditions: baseline, visual low/high (background characters), auditory low/high (background sounds), and mental low/high (0-back/2-back tasks), in random order. Outcome measures included MDE (0-100), task performance (products placed in the cart), mean heart rate (from electrocardiography), head-stillness duration, and gaze duration on areas of interest (via integrated eye-tracking). Two-tailed paired-samples *t* tests with Bonferroni correction (α=.008) were supplemented by linear mixed-effects models.

**Results:**

The mental high condition produced the largest effects: MDE increased relative to baseline (mean difference [*M*_diff_] 52.73, 95% CI 44.01-61.44; t_21_=12.58; *P*<.001), performance decreased (*M*_diff_ –8.00 products, 95% CI –9.57 to –6.43; t_21_=–10.58; *P*<.001), head-stillness duration increased (*M*_diff_ 13.54 s, 95% CI 9.94-17.14; t_21_=7.83; *P*<.001), and gaze on the shopping list increased (*M*_diff_ 9.89 s, 95% CI 5.36-14.43; t_21_=4.54; *P*<.001). Auditory high demands increased MDE (*M*_diff_ 17.05, 95% CI 9.15-24.94; t_21_=4.49; *P*<.001) without significantly affecting performance. Visual high demands redistributed gaze away from the main shelves (*M*_diff_ –6.11 s, 95% CI –8.70 to –3.52; t_21_=–4.91; *P*<.001) without reducing performance. Heart rate increased under mental low demand (*M*_diff_ 4.02 bpm, 95% CI 1.69-6.34; t_21_=3.59; *P*=.002); the mental high effect did not reach corrected significance in the paired-samples test (*P*=.02), possibly due to reduced physical activity in this condition. Under high cognitive load, participants spent more time checking the shopping list and showed greater head-stillness, consistent with task-serialization and memory-offloading strategies.

**Conclusions:**

Cognitive demands strongly impacted workload, performance, and behavior, while sensory demands affected attention distribution and perceived workload, with no significant performance decrements at the Bonferroni-corrected threshold. By comparing 2 demand levels against a no-demand baseline and incorporating gaze and head-movement measures not previously examined, this study extends prior work in this domain. The findings suggest IVR environments can incorporate sensory richness without large performance reductions in unimpaired young adults, and that behavioral strategies under cognitive load may support real-world workload monitoring and adaptive task adjustment, pending clinical validation.

## Introduction

Immersive virtual reality (IVR) visualized through head-mounted displays (HMDs) is increasingly used for training and shows promise for neurorehabilitation [1-3]. IVR allows the creation of personalized, simulated real-life environments that can support the intensive and safe practice of task-specific movements, for example for users with neurological conditions such as stroke [4] and Parkinson disease [5]. These characteristics are especially relevant for brain-injured individuals, as neurorehabilitation evidence encourages high-dose, task-specific, personalized motor practice [6-8]. However, conventional training environments may lack adequate stimulation or appropriate difficulty [9], whereas IVR can provide stimulation calibrated to individual capacity [4,10]. This adaptability is particularly relevant for individuals with sensory sensitivity after acquired brain injury, for whom adjusting task and stimulus complexity may help optimize the level of challenge, avoid over- or underexertion, and maintain focus on core tasks.

Previous research has shown that environmental complexity and additional sensory stimuli in immersive virtual environments can influence users' mental workload [11] and task performance [12]. Additionally, research on cognitive workload under noise conditions suggests that noise may require additional cognitive resources to maintain performance on a primary task, and that psychophysiological measures can help quantify this compensatory workload depending on the type of auditory stimulation and cognitive task involved [13]. Cucinella et al [14] investigated the impact of visual, auditory, and cognitive demands on unimpaired users’ mental demand and effort (MDE) and physiological activity during a grocery shopping task in an immersive virtual supermarket. Under conditions of added visual (background characters), auditory (background noise), and cognitive demands (concurrent arithmetic), the cognitively demanding task competed with the shopping task's cognitive requirements, substantially increasing completion time, heart rate, and MDE relative to sensory-only conditions, consistent with competition for shared central/working-memory resources [15]. However, that study did not examine higher levels of visual and auditory demands, did not include a no-demand baseline, and did not record behavioral measures such as gaze direction. It therefore remains unclear how graded sensory demands influence users' behavioral responses during IVR task execution. Such behavioral data could give trainers insight into how users engage with tasks, where they struggle, and what strategies they adopt.

Therefore, we experimented with 22 unimpaired participants (ie, without known sensorimotor, cognitive, visual, or auditory impairments) to examine how 2 levels of visual, auditory, and cognitive demands influence MDE, task performance, gaze behavior, and head movements during a virtual shopping task in an immersive virtual supermarket (modified from Cucinella et al [14]). The design also included a baseline condition without added demands. The virtual supermarket replicates an activity of daily living used in IVR for cognitive and functional assessment [16,17] and in consumer research on shopping behavior [18-20]. Here, the virtual environment serves to establish a baseline understanding of how sensory and cognitive demands affect performance, workload, and behavior in this specific IVR task and population, rather than consumer behavior. We used the NASA task load index (NASA-TLX) questionnaire to assess MDE and recorded task performance (total number of products placed in the cart) [21,22]. Although traditionally associated with physical demands [23], heart rate [24,25] also responds to mental workload, warranting investigation in this context. We also recorded head-stillness duration, since increased mental demands are associated with altered head-hand coordination [26], and gaze behavior on specific areas of interest (AOIs), given that mental demands influence fixation patterns and durations [27-29].

We tested the following hypotheses: (1) compared to baseline (no added demands), high visual and auditory demands primarily distract attention, leading to more head movements and gaze toward nontask-relevant elements, and (2) mental demands negatively affect task execution, increasing MDE, reducing performance, and producing behavioral adaptations such as reduced head movement and increased focus on the shopping list.

## Methods

### Participant Characteristics

A total of 22 unimpaired participants took part in the experiment. The participants were all university students aged between 23 and 27 years without known sensorimotor, cognitive, visual, or auditory impairments, including 11 women and 11 men. Handedness was not an inclusion criterion; however, task interaction was restricted to the participant's right hand due to the placement of electrodermal activity (EDA) sensors on the left hand. While EDA data were recorded, these were excluded from the analysis as hand movements caused frequent data corruption for most participants.

### Sampling Procedures

Participants were recruited from TU Delft, the Netherlands, via word of mouth (convenience sampling). The experiment was conducted in a laboratory at TU Delft between October 27, 2023, and November 3, 2023.

### Sample Size

The sample size of 22 participants was based on our previous study [14], which used a comparable within-subjects design and task with 24 participants. No formal a priori power analysis was conducted. A post-hoc sensitivity analysis indicated that, at the Bonferroni-corrected significance level (α=.008) and 80% power, the design could reliably detect within-subject effects of |d_z_|≥0.81 in a 2-tailed paired *t* test (*df*=21).

### Ethical Considerations

The study was approved by the Human Research Ethics Committee (HREC) of TU Delft under approval number 3417. All participants provided written informed consent for participation in the study and for the collection and use of their data, including physiological recordings (electrocardiography [ECG], eye-tracking, and EDA), audio and screen recordings, and questionnaire responses. Participant data were de-identified using numerical codes, signed consent forms were stored separately in a locked cabinet, and only the research team had access to the study data. Participants received a 15-euro gift card as compensation. No adverse events, such as motion sickness requiring session termination, were reported. No identifiable images of individual participants are included in the manuscript or supplementary material.

### Masking

Participants were not informed of the specific condition before each trial, except for the mental low and mental high conditions, which required pretrial instructions for the concurrent *n*-back task. Visual and auditory manipulations became apparent once the trial began. The researcher administering the experiment was aware of all condition assignments. No formal blinding of outcome assessors was implemented, as all dependent measures were recorded automatically via the HMD, eye tracker, and ECG system.

### Instrumentation

The experiment was conducted in a laboratory room with a walkable area of approximately 5.0 m × 2.5 m (12.5 m^2^), providing enough space for participants to perform physical movements without risk of collision.

The experimental setup featured an HTC VIVE Pro Eye HMD. This HMD provided a 110-degree field of view, dual OLED screens with a resolution of 1440 × 1600 pixels per eye (2880 × 1600 combined), and integrated eye-tracking with a native sampling rate of 120 Hz. The setup also included 1 HTC VIVE tracked controller (HTC VIVE Pro Controller 2.0) and 2 base stations. Eye-gaze data, participants' actions, and HMD tracking data were all logged once per rendered frame at approximately 86 Hz and interpolated to a constant frequency of 100 Hz using nearest-neighbor interpolation. Each frame, Unity’s raycasting engine assigned the gaze to a single AOI based on the nearest object intersected by the gaze ray. No temporal smoothing was applied to AOI labels in the statistical analysis. Frames with gaze tracking loss, identified by a null ray direction vector, were classified as “Tracking loss”, a category separate from the 4 AOIs and from “Other.” “Other” comprises gaze on nontask elements (eg, characters) and valid gaze rays landing outside all AOIs. This distinction is described further in data diagnostics.

We developed the virtual environment in Unity (v2021.3.11f1) and displayed it via the SteamVR plugin (v2.7.3). Eye-tracking data were collected within Unity using the integrated Tobii XR and SRanipal SDKs. The experiment used a virtual supermarket environment modified from Cucinella et al [14], populated with product and character assets (eg, onions, bread, NPC populator, low poly animated people packages) from the Unity Asset Store. Auditory stimuli (soundtracks from Envato Elements, Pixabay) were delivered through the HTC VIVE Pro Eye headphones. The participant's view was screen-recorded using Windows Xbox Recorder.

ECG data were acquired at 1024 Hz via Bluetooth using BioTrace+ software (MindMedia, NeXuS-4) with electrodes in a Lead II configuration. The experiment ran within the Unity Editor on a Windows 10 64-bit PC (AMD Ryzen 9 5900X CPU @ 3.70 GHz, 32 GB DDR4 RAM, NVIDIA GeForce RTX 3080 GPU).

### Virtual Grocery Shopping Task

**Figure 1:**
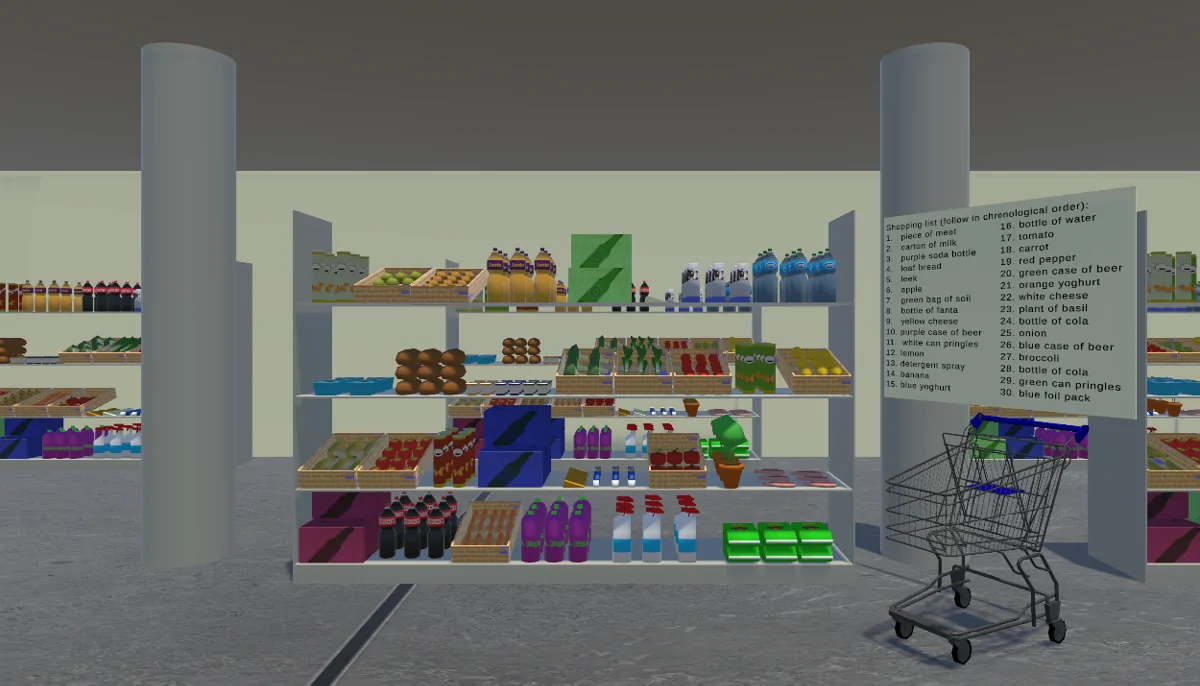
Overview of the immersive virtual supermarket task environment used in this study, showing the four shelves, the shopping list, and the cart, from a remote perspective.

Participants engaged in a virtual shopping task, collecting products from 4 shelves in front of them according to a sequentially ordered list of 30 products, and placing them in a cart (Figure 1). Each trial lasted 122.4 seconds.

To investigate how external task demands influence task performance, perceived workload, physiological activity, and behavioral strategies, we varied the order and locations of products across trials. The 4 identical, equidistant shelves contained 33 or 34 products (6-11 per shelf), depending on the condition. Each shopping list contained 30 items distributed across 4 shelves; in some conditions, 1 or 2 products appeared twice on the list. The listed subset, list order, and product-shelf assignments varied across conditions but were fixed across participants within a condition. Consecutive list items were grouped in sets of 4, 1 per shelf. This constraint held for positions 1-16 in all conditions and for positions 1-28 in baseline. With a mean of 19 products collected at baseline, the majority of task execution fell within the balanced range.

Participants could move freely and used the controller button (hold to grab, release to drop) to interact with products via a visible virtual right hand (no virtual body was shown). In a within-subjects design, all participants performed 7 conditions, whose order was randomized per participant.

### Conditions and Design

The experiment included 7 conditions, summarized in Tables 1 and 2. The baseline condition consisted of the shopping task without any additional stimuli and was used as the reference condition for all comparisons. The 6 experimental conditions added visual, auditory, or cognitive demands on top of the same core shopping task; the core task procedure was the same across all conditions.

**Table 1:** Descriptions of visual demand manipulations across the visual conditions in the immersive virtual supermarket during the shopping task.

Factor	Visual low	Visual high
Character type	Only casual characters	Also, unusual and remarkable characters
Character actions	Only characters walking, waving, and looking at the participant	Also, doing additional moves, such as dancing
Events	Only characters	Also, noncharacter elements as visual distractors, such as a flashing alarm
Location	Only characters behind the main shelves, at least 4 meters from the participant’s starting point	Also next to the main shelves, at least 2 meters from the participant’s starting point
Examples	Casually dressed characters walking, waving, or looking at the user	Also, unusually dressed characters, such as clowns and pilots, flashing alarms, and characters dancing, jumping, and calling

**Table 2:** Description of auditory demand manipulations across the auditory conditions in the immersive virtual supermarket during the shopping task.

Factor	Auditory low	Auditory high
Motion	Only static sounds or moving at a walking pace	Also, moving at a running or driving pace
Duration	Continuous audio fragments	Also, short and repeating audio fragments
Location	At least 5 meters from the participant’s starting point	At least 2 meters from the participant’s starting point
Examples	Checkout desk sounds, beeps, a couple arguing, crowd conversations, walking footsteps in front of the participant	Also, sirens from police cars, a nearby person calling to the participant, running footsteps behind the participant, a truck in reverse, and bottles falling

**Figure 2:**
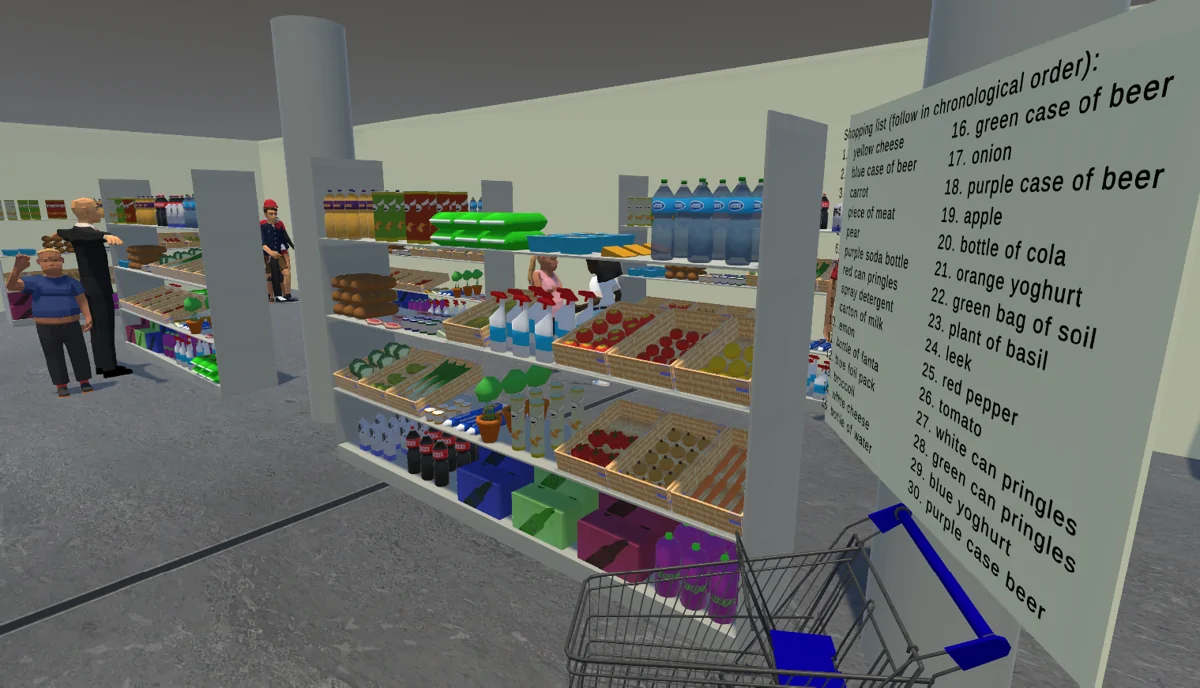
The immersive virtual supermarket task environment during the visual low condition, shown from a remote perspective. Casually dressed characters were positioned behind the main shelves, at least 4 meters from the participant's starting position.

**Figure 3:**
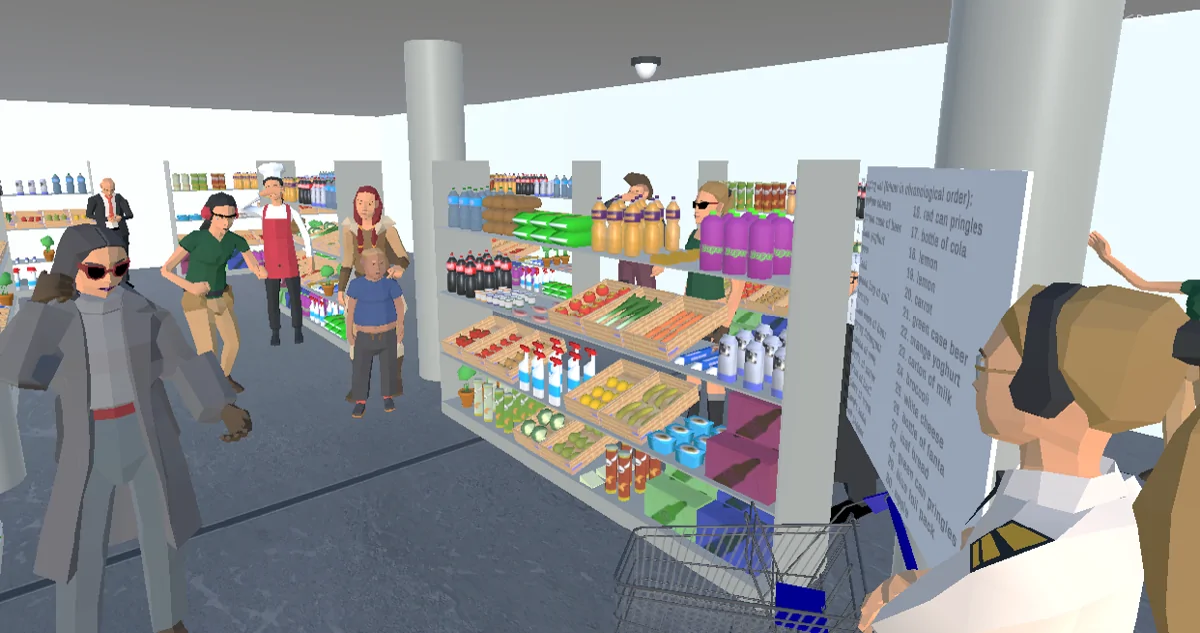
The immersive virtual supermarket task environment during the visual high condition, shown from a remote perspective. Additional characters with distinct appearances were positioned closer to the participant, compared to the visual low condition.

1. Baseline: it consisted only of the experimental task. No extra stimuli were presented.
2. Visual low: the scene included 8 idle characters (eg, standing in the environment) and 22 moving characters (eg, spawning, following a trajectory, and disappearing). Eight of these 22 moving characters performed specific actions, such as sending messages to the participant by waving or using a thumbs-up gesture. Some moving characters navigated freely through walkable areas delineated by the shelves. All the characters were casually dressed. Their actions took place behind the shelves and in the neighboring aisle. Characters in the same aisle as the main shelves remained idle and were positioned at least 4 meters from the participant’s starting position (Figure 2).
3. Visual high: in this condition, 11 idle and 44 moving characters were presented; 28 of the moving characters performed specific actions. This condition differed from the Visual low condition regarding character types, actions, events, and locations. Characters were positioned closer (at least 2 meters from the participant’s initial position) and had distinct appearances (eg, policeman, clown, viking, and chef). Additionally, 2 alarm lights were installed on the ceiling above the main shelves (Figure 3).
4. Auditory low: participants experienced spatial stereo audio delivered via the HMD’s headphones. The audio sources were dynamic: their perceived direction rotated in sync with the participant’s head movements (tracked via the HMD), and their volume attenuated based on the virtual distance between the participant and the sound source. To achieve this in the Unity environment, the resonance audio listener plugin was used. The sound field represents full 360-degree spatial audio by encoding sound waves on a virtual sphere around a listener. The auditory environment included 2 different footstep sound sequences moving along predefined trajectories and 3 stationary ambient sounds. The footstep sounds simulated a standard walking pace and originated from virtual locations kept at least 5 meters away from the participant’s starting position.
5. Auditory high: spatial stereo sounds were played, consisting of several components: 3 footstep trajectories, 5 continuous idle sounds, 2 intermittent idle sounds (repeating with breaks), and 6 incidental sounds (eg, short fragments originating from 2 meters away). The footstep sounds simulated movement at both walking and running paces. The perceived sound sources were at least 2 meters from the participant’s initial position.
6. Mental low: a 0-back task was added to the main task. Participants listened through the HMD's headphones to a prerecorded voice, giving 4 sets of random single-digit numbers and then repeating aloud the last number they heard. The first 3 sets consisted of 10 numbers, but the fourth had only 8. Digits were presented with 2.5 seconds between the start of 1 digit and the start of the next. The 0-back task was used rather than a neutral control (eg, button pressing) to approximate the sensory and motor requirements of the 2-back task (see below).
7. Mental high: a 2-back task was added to the main task. Similar to the 0-back task, participants repeated aloud the number they heard 2 numbers earlier through the HMD’s headphones. Again, the first 3 sets consisted of 10 numbers, but the fourth had only 8.

All experimental manipulations were delivered as programmed in Unity. No technical failures affecting the visual, auditory, or cognitive manipulations were observed during data collection.

### Measures and Covariates

The following dependent measures were calculated per trial:

1. Self-reported MDE (0 to 100): the NASA-TLX [21] was administered after each trial. As in [14], only scores from the MDE items were taken since the questions consider the mental or cognitive aspects of the task, which are the focus of this study. Therefore, our current analysis focuses on the average of 2 items: mental demand (how mentally demanding was the task?) and effort (how hard did you have to work to accomplish your level of performance?). Participants rated the 2 items on a 100-point interval scale in 5-point increments, ranging from 0 (low) to 100 (high).
2. Performance: the total number of collected products that are placed in the shopping cart.
3. Heart rate (beats per minute, [bpm]): we used a MATLAB implementation of the Pan-Tompkins QRS Detector, as developed by Sedghamiz [30], to extract heartbeats from the ECG signal. The method involves a sequence of signal processing steps designed to identify the QRS complexes in an ECG signal. Interbeat intervals (IBIs) were screened for artifacts (see Data Diagnostics). The mean heart rate for a particular trial was calculated as 60 divided by the mean of the remaining valid IBIs.
4. Head-stillness duration: we quantified head-stillness duration, defined as the total time angular head speed remained below a threshold of 0.2 rad/s (11.5 deg/s). This threshold is a heuristic, but informed by amplitude and velocity criteria previously used to identify and classify head movements in eye-head coordination studies [31,32]; it was chosen to capture periods in which the head was not engaged in active reorientation. To calculate this measure, we first smoothed the quaternion head orientation data using a geometry-aware filter: quaternions were mapped to their logarithmic representation (axis-angle vectors), filtered using a zero-phase, 2nd-order Butterworth lowpass filter (2 Hz cutoff), and then converted back to orientations. Angular head speed was calculated based on the rate of orientation change between consecutive time points of the smoothed quaternions, measured as twice the arccosine of the absolute value of the dot product of each pair of consecutive quaternions. The total head-stillness duration represents the cumulative time in seconds of all sampling intervals for which the computed angular speed satisfied the stillness threshold.
5. Gaze duration on AOIs: we defined several AOIs within the environment: the shopping list, the main shelves, the shopping cart, and human characters. Gaze data from the human characters AOI and outside designated AOIs were combined into a single category labeled “Other.” The time spent gazing at the other category might reflect moments of distraction or difficulty maintaining focus on task-relevant elements. In contrast, increased gaze time on the shopping list may reflect that participants used it during task execution, which is consistent with previous research showing that offloading cognition onto external resources can support task performance [33-35].

### Data Collection

After providing informed consent upon arrival, participants received instructions for the shopping task. They learned the primary goal was to read products from a list, retrieve them from designated shelves using the controller in their right hand (with specific button presses for grab/release), and place them into the shopping cart. Participants were asked to keep their left hand still, avoid throwing products, follow the list chronologically, and not search outside the specified areas. It was pointed out that the list was intentionally long and not meant to be completed, and that they should not rush. They were also informed that products would vanish from the cart shortly after placement, advised about potential motion sickness, and reminded that they could withdraw at any time.

A familiarization session before putting on the HMD specifically addressed potentially confusing products. Examples given were a blue foil pack, a Fanta bottle labeled “Santa,” and leeks, which the researchers noted might be an unfamiliar term for Dutch participants. The need to pay attention to color was also highlighted, using different colored yogurts as an example.

Participants then self-applied ECG electrodes (lead II configuration), and the researcher started screen-recording the HMD view. Once the HMD was fitted, the eye tracker was calibrated. During calibration, the virtual height was set to 185 cm to ensure consistent visibility across participants, particularly for shorter individuals viewing top-shelf products.

An initial practice trial of approximately 1.5 minutes allowed participants to acclimatize to the environment and controls without task demands. Subsequently, participants underwent 7 experimental trials, each featuring a different condition (eg, visual low and auditory high) presented in random order. Eye-tracking recalibration occurred before each trial. For the n-back trials, participants were briefed on the concurrent task requirements, informed of its equal priority with shopping, and provided an audio example.

Posttrial procedures involved removing the HMD and completing a condition-specific NASA-TLX questionnaire via Qualtrics. A final NASA-TLX for item weighting was completed after the last trial, although these weights were not used in the analysis.

The experiment concluded with a gift card compensation. Each participant’s session took roughly 75 to 90 minutes.

### Data Diagnostics

No participants were excluded after data collection. Heart rate data were unavailable for 3 out of 154 trials (3/154, 1.9%; participant 22: visual low and visual high; participant 12: auditory low), likely due to electrode displacement. Given the small proportion of missing data, no imputation was performed; these trials were excluded pairwise for heart rate analyses, resulting in 20 degrees of freedom for the affected comparisons. Little’s missing completely at random (MCAR) test, conducted across the 8 trial-level dependent measures (MDE, performance, heart rate, head-stillness duration, and the 4 gaze AOI durations), did not reject the MCAR assumption (χ^2^_7_=5.26; *P*=.63). IBIs were screened for artifacts using the Berntson et al [36] criterion with the placement parameter set to 0.75 (ie, 75% from the maximum expected difference toward the minimum artifact difference), resulting in the rejection of 902 of 27,063 IBIs (3.3%) across all trials. One participant had substantially higher rejection rates in individual trials (up to 66.5%), though their remaining valid IBIs were retained for analysis.

Gaze tracking loss occurred on a mean of 5.3% of frames per trial (SD 2.8%; mean 561 of 10,516 frames per trial at the ~86 Hz logging rate, across 154 trials). In total, 86,364 of 1,619,535 frames (86,364/1,619,535, 5.3%) were affected. These frames were classified as Tracking loss, separate from the other gaze category, which includes only gaze with a valid ray direction that did not intersect any AOI. Tracking loss was similar across conditions (range 4.7%-5.2%) except in the mental high condition (7.8%), where it was significantly elevated relative to baseline (baseline: mean 5.83, SD 2.72 s; mental high: mean 9.41, SD 4.38 s; mean difference 3.58 s, 95% CI 1.84-5.31; t_21_=4.29; *P*<.001). An episode-level analysis based on episode duration showed that across all conditions, 4213 of 7339 tracking-loss episodes (4213/7339, 57%) lasted 100-400 ms, consistent with the typical duration of spontaneous eye blinks. Under mental high, the elevated tracking loss reflected increases across multiple duration categories: typical blinks (100-400 ms) rose from 4.56 seconds to 6.39 seconds per trial (t_21_=3.64; *P*=.002), long blinks (400-1000 ms) rose from 0.37 seconds to 1.51 seconds (t_21_=3.26; *P*=.004), and very long gaps (>1000 ms), absent in all other conditions, contributed 0.47 seconds per trial. By contrast, the duration of short jitter episodes (<100 ms) did not increase significantly (mental high: 1.07 s vs baseline: 0.98 s; t_21_=0.73; *P*=.48).

No statistical outliers were removed. The degree of nonnormality of the paired difference scores (condition minus baseline) was assessed descriptively. Of 48 comparisons, 31 had |skewness| < 0.5, 8 had |skewness| between 0.5 and 1.0, and 9 had |skewness| > 1. The most skewed comparisons involved gaze on other elements (auditory low: 3.06; mental low: 2.24; mental high: 2.06) and heart rate, auditory high (2.23). In each case, the skew was traced to a single participant with an extreme difference score; excluding the respective participant did not change the significance of any comparison for gaze on other elements or heart rate. The 2-tailed paired-samples *t* test is relatively robust to outlier contamination [37].

### Analytic Strategy

To quantify the size of the differences between conditions, we calculated Cohen *d* (measuring the overall difference between conditions) and Cohen *d_z_* (measuring the degree of change within participants). Since we performed 6 comparisons against the baseline, we applied a Bonferroni correction, adjusting our significance threshold to *P<*.008 (α=.05/6) to control the risk of false positives. As a robustness check, we fitted linear mixed-effects models for each dependent measure (Multimedia Appendix 1). Model B corrects for presentation order (trial position 1-7) as a linear covariate to control for learning or fatigue effects across the experimental session. Effect sizes were computed following the convention of the emmeans package [38]. Full results are reported in Multimedia Appendix 1.

### Exploratory Behavior Analysis

While the above dependent measures provide global indications of participants’ behaviors, understanding how they adapted their strategies requires examining their behaviors more closely. Gaze behavior on AOIs, task performance, and product-grabbing actions were used to create “behavioral charts” for a representative participant in the baseline, visual high, auditory high, and mental high conditions. To reduce high-frequency jitter in the visualized AOI labels, a 1-dimensional mode filter with a window size of 19 samples (190 ms) was applied. This chart shows how either the sensory or the cognitive demands affected a participant's shopping routine of reading the shopping list, locating products on the main shelves, and grabbing and placing them in the cart. The x-axis represents the elapsed time within the trial in seconds. The y-axis categorizes gaze targets (ie, list, main shelves, and cart) and activities (ie, grabbing products). These actions are visualized in the form of colored bars of variable length, corresponding to the duration of each behavior. Additionally, we analyzed the percentage of time spent looking at the shopping list as a function of normalized time between consecutive product placements to examine differences in the participants’ strategies compared to the baseline.

## Results

### Differences Between the Six Experimental Conditions and Baseline

Table 3 shows descriptive statistics (ie, mean, SD) calculated over the 22 participants and the 2-tailed paired-samples *t* test results for each dependent measure (ie, MDE, performance, heart rate, head-stillness duration, gaze duration). We also included Cohen *d* and the within-subject effect size Cohen *d_z_*, as well as the mean difference from baseline with 95% CIs.

**Table 3:** Means (SD), results of paired-sample *t* tests, effect size measures, and mean differences from baseline to the corresponding condition in the immersive virtual environment during the shopping task with unimpaired participants (M_diff_) with 95% CIs, for the dependent measures, namely mental demand and effort, performance, heart rate, head-stillness duration, and gaze duration on areas of interest.

		Mean (SD)	*t* test (*df*)	*P* value	*d*	*d*_*z*_	*M*_diff_	95% CI
Self-reported mental demand and effort (MDE) (0 to 100)								
	Baseline	22.05 (16.56)	—^b^	—	—	—	—	—
	Visual low	21.59 (14.71)	−0.20 (21)	.84	−0.03	−0.04	−0.45	−5.23 to 4.32
	Visual high	27.61 (14.87)	1.97 (21)	.06	0.35	0.42	5.57	−0.30 to 11.43
	Auditory low	27.84 (15.95)	2.10 (21)	.05	0.36	0.45	5.80	0.07 to 11.52
	Auditory high	39.09 (20.17)	4.49 (21)	<.001	0.92	0.96	17.05	9.15 to 24.94
	Mental low	41.93 (15.41)	4.49 (21)	<.001	1.24	0.96	19.89	10.67 to 29.11
	Mental high	74.77 (13.34)	12.58 (21)	<.001	3.51	2.68	52.73	44.01 to 61.44
Performance (total number of products placed in the cart)								
	Baseline	18.68 (2.70)	—	—	—	—	—	—
	Visual low	19.73 (4.86)	1.29 (21)	.21	0.27	0.28	1.05	−0.64 to 2.73
	Visual high	18.55 (3.66)	−0.19 (21)	.85	−0.04	−0.04	−0.14	−1.66 to 1.38
	Auditory low	19.18 (3.17)	0.76 (21)	.46	0.17	0.16	0.50	−0.87 to 1.87
	Auditory high	17.50 (4.24)	−1.63 (21)	.12	−0.33	−0.35	−1.18	−2.69 to 0.33
	Mental low	18.23 (4.23)	−0.54 (21)	.59	−0.13	−0.12	−0.45	−2.21 to 1.30
	Mental high	10.68 (3.26)	−10.58 (21)	<.001	−2.68	−2.26	−8.00	−9.57 to −6.43
Mean heart rate (bpm)								
	Baseline	87.46 (12.78)	—	—	—	—	—	—
	Visual low	87.33 (14.71)	−0.40 (20)	.70	−0.02	−0.09	−0.27	−1.72 to 1.17
	Visual high	86.40 (14.66)	−1.14 (20)	.27	−0.09	−0.25	−1.21	−3.41 to 0.99
	Auditory low	86.49 (13.26)	−0.50 (20)	.62	−0.03	−0.11	−0.43	−2.24 to 1.37
	Auditory high	87.97 (15.95)	0.48 (21)	.64	0.04	0.10	0.51	−1.69 to 2.70
	Mental low	91.47 (15.26)	3.59 (21)	.002	0.29	0.77	4.02	1.69 to 6.34
	Mental high	90.28 (13.53)	2.45 (21)	.02	0.21	0.52	2.82	0.43 to 5.21
Head-stillness duration (s)								
	Baseline	14.89 (5.82)	—	—	—	—	—	—
	Visual low	15.26 (6.14)	0.39 (21)	.70	0.06	0.08	0.36	−1.59 to 2.31
	Visual high	14.71 (5.93)	−0.23 (21)	.82	−0.03	−0.05	−0.19	−1.93 to 1.56
	Auditory low	14.98 (6.17)	0.08 (21)	.94	0.01	0.02	0.08	−2.19 to 2.35
	Auditory high	16.13 (8.11)	0.94 (21)	.36	0.17	0.20	1.23	−1.49 to 3.96
	Mental low	15.72 (5.96)	1.16 (21)	.26	0.14	0.25	0.83	−0.66 to 2.31
	Mental high	28.44 (10.77)	7.83 (21)	<.001	1.56	1.67	13.54	9.94 to 17.14
Gaze duration on the shopping list (s)								
	Baseline	31.21 (6.10)	—	—	—	—	—	—
	Visual low	31.70 (5.67)	0.68 (21)	.51	0.08	0.14	0.50	−1.03 to 2.02
	Visual high	31.43 (3.93)	0.23 (21)	.82	0.04	0.05	0.22	−1.74 to 2.17
	Auditory low	33.59 (4.66)	2.29 (21)	.03	0.44	0.49	2.38	0.22 to 4.54
	Auditory high	32.25 (5.71)	1.23 (21)	.23	0.18	0.26	1.04	−0.72 to 2.80
	Mental low	36.17 (6.82)	4.28 (21)	<.001	0.77	0.91	4.96	2.55 to 7.37
	Mental high	41.10 (8.60)	4.54 (21)	<.001	1.33	0.97	9.89	5.36 to 14.43
Gaze duration on the main shelves (s)								
	Baseline	73.53 (6.67)	—	—	—	—	—	—
	Visual low	70.70 (5.54)	−2.43 (21)	.02	−0.46	−0.52	−2.83	−5.26 to −0.41
	Visual high	67.42 (5.38)	−4.91 (21)	<.001	−1.01	−1.05	−6.11	−8.70 to −3.52
	Auditory low	70.90 (6.38)	−2.03 (21)	.06	−0.40	−0.43	−2.63	−5.33 to 0.07
	Auditory high	72.77 (5.17)	−0.68 (21)	.50	−0.13	−0.15	−0.77	−3.10 to 1.57
	Mental low	69.74 (6.79)	−3.35 (21)	.003	−0.56	−0.71	−3.80	−6.15 to −1.44
	Mental high	60.29 (10.38)	−5.28 (21)	<.001	−1.52	−1.13	−13.24	−18.45 to −8.03
Gaze duration on the cart (s)								
	Baseline	8.91 (2.50)	—	—	—	—	—	—
	Visual low	9.01 (3.57)	0.14 (21)	.89	0.03	0.03	0.09	−1.33 to 1.52
	Visual high	8.18 (3.03)	−1.47 (21)	.16	−0.26	−0.31	−0.73	−1.76 to 0.30
	Auditory low	8.15 (2.84)	−1.72 (21)	.10	−0.29	−0.37	−0.77	−1.69 to 0.16
	Auditory high	8.50 (3.20)	−0.79 (21)	.44	−0.14	−0.17	−0.41	−1.49 to 0.67
	Mental low	7.43 (2.66)	−2.90 (21)	.009	−0.57	−0.62	−1.48	−2.55 to −0.42
	Mental high	7.62 (2.82)	−1.86 (21)	.08	−0.49	−0.40	−1.29	−2.73 to 0.15
Gaze duration on other elements (s)								
	Baseline	2.92 (0.98)	—	—	—	—	—	—
	Visual low	5.21 (2.19)	4.93 (21)	<.001	1.35	1.05	2.29	1.32 to 3.25
	Visual high	9.70 (4.35)	7.93 (21)	<.001	2.15	1.69	6.78	5.00 to 8.56
	Auditory low	3.51 (3.19)	0.83 (21)	.42	0.25	0.18	0.58	−0.88 to 2.05
	Auditory high	3.13 (1.29)	0.88 (21)	.39	0.18	0.19	0.21	−0.28 to 0.70
	Mental low	2.91 (1.64)	−0.04 (21)	.97	−0.01	−0.01	−0.01	−0.78 to 0.75
	Mental high	3.98 (3.23)	1.49 (21)	.15	0.44	0.32	1.06	−0.42 to 2.54

^a^*P* value<.008 (.05/6) was considered statistically significant; *t*, *P* value, *d_z_*, *M*_diff_, and 95% CI are based on paired differences. Degrees of freedom are 21 for all comparisons except the three heart rate comparisons affected by missing data (visual low, visual high, and auditory low: *df*=20).

^b^Not applicable.

The mental high condition produced the largest and most consistent effects, with statistically significant differences from baseline on all dependent measures except heart rate, gaze on the cart, and gaze on other elements (Table 3). The mental low condition also showed significant effects on MDE, heart rate, gaze on the shopping list, and main shelves.

In the 2-back task, 38 items were presented, of which the first 2 items in each block were not scorable, yielding 30 scorable items. Mean accuracy was 19.1 (SD 6.3) out of 30 scorable items (19.1/30, 64%; scored for 21 of 22 participants; audio recordings failed for 1 participant), confirming that the task was cognitively demanding.

**Figure 4:**
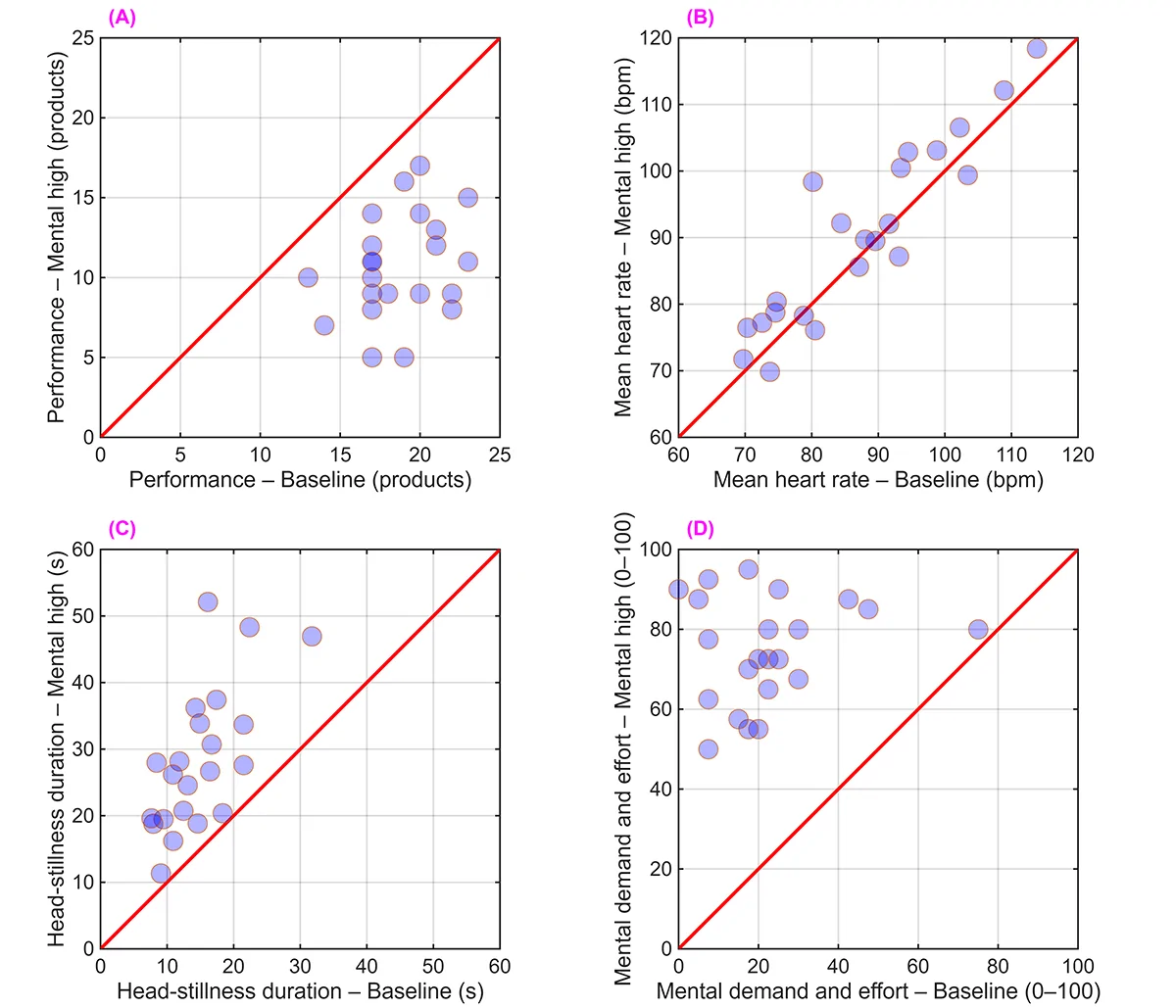
Scatter plots of four dependent measures (A: performance; B: mean heart rate; C: head-stillness duration; D: mental demand and effort [MDE]), comparing scores in the baseline condition to those in the mental high condition during the immersive virtual shopping task. Each marker represents one participant. Transparent markers are used to see overlapping data points more clearly. Red diagonals indicate equal values for both conditions.

For some dependent measures, *d_z_* was larger than *d*, notably heart rate (Figure 4B). Among the sensory conditions, only the auditory high condition significantly increased MDE, and only the visual conditions significantly redistributed gaze (for visual high: less on main shelves; for visual low and visual high: more on other elements, ie, likely the characters). No sensory condition significantly affected performance.

### Participants’ Behaviors

**Figure 5:**
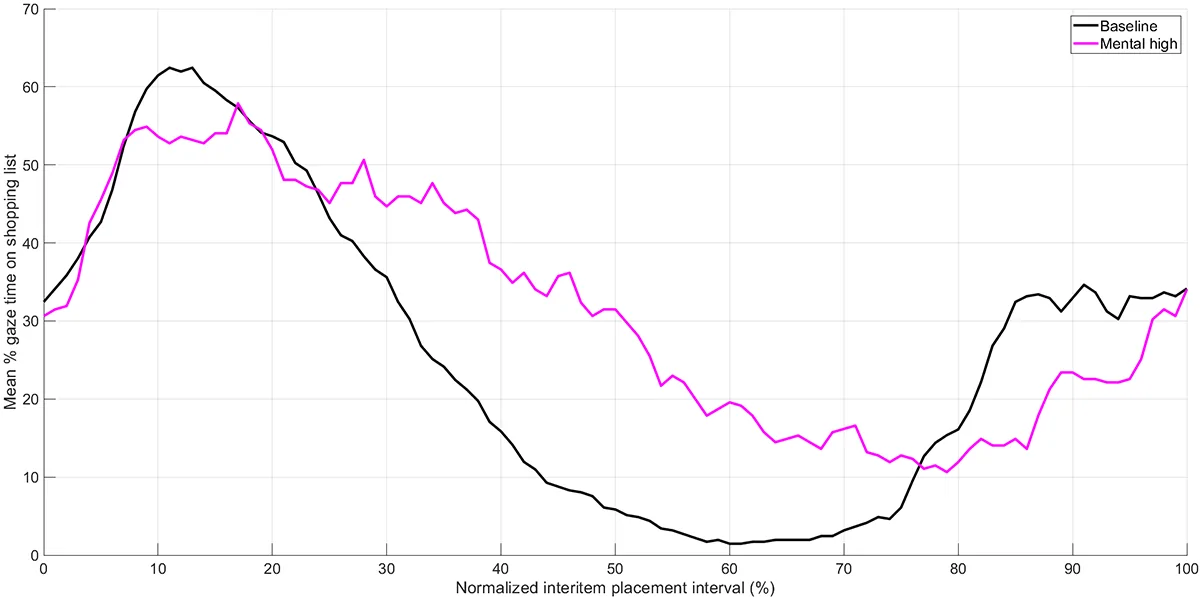
Mean percentage of gaze time on the shopping list as a function of normalized time between consecutive product placements, comparing the baseline and mental high conditions during the immersive virtual shopping task. Data are averaged across all unimpaired participants and interplacement intervals.

Figure 5 illustrates the average percentage of time participants gazed at the shopping list during the interval between placing consecutive products in the cart. Participants during the baseline condition showed a peak of attention toward the shopping list early in the interval, which then dropped, indicating they likely encoded the next product and focused on searching for the product on the shelves. Conversely, participants in the mental high condition maintained a relatively higher percentage of gaze time on the shopping list throughout the mid-to-late stages of the interval, indicating more frequent rechecking or consultation under increased mental demand.

**Figure 6:**
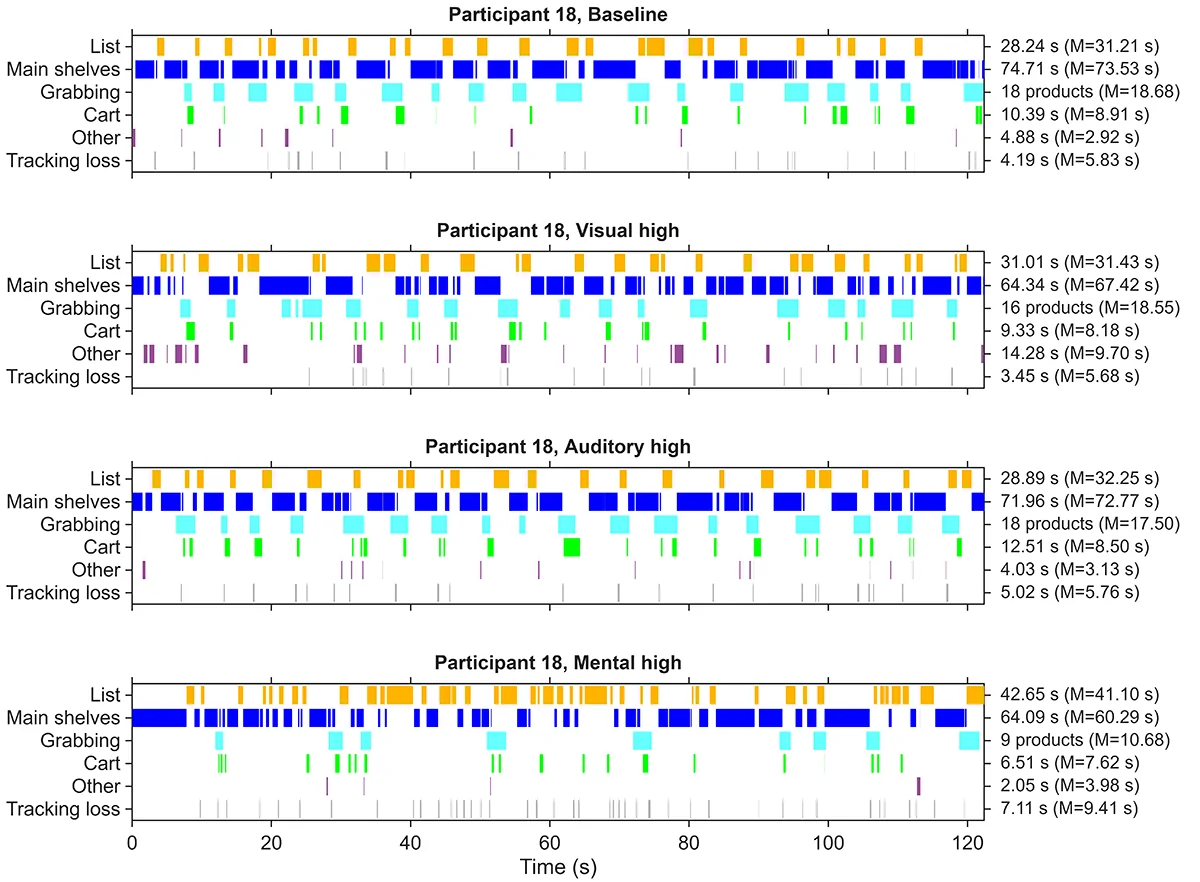
Behavioral charts for the unimpaired young participant 18 during the immersive virtual shopping task at baseline, visual high, auditory high, and mental high conditions of the within-subjects experimental study. Colored bars indicate gaze on the shopping list (orange), main shelves (blue), cart (green), other elements (purple), eye-tracking loss (gray), as well as product grabbing (cyan), over the 122.4-second trial. Values on the right show the participant's gaze durations and products collected, with the mean of participants (M) in parentheses.

Behavioral charts in Figure 6 show, over time, the gaze patterns on the shopping list, the main shelves, and the shopping cart, as well as the time products were held, for a single participant (Participant 18, selected because their baseline performance was close to the group mean) in the baseline, visual high, auditory high, and mental high conditions.

Observations from the behavioral charts indicate that participants in the baseline condition generally followed a consistent strategy: first checking the shopping list to identify the target product, then locating it on the main shelves. After locating the product, participants grabbed it and placed it in the shopping cart. However, we observed some variations: some participants reduced the frequency of checking the shopping list, collecting multiple products consecutively before revisiting it, while others checked the list for the next product while placing the previously collected product in the cart. As illustrated in Figure 6, this general strategy remained largely the same in the auditory high condition, with gaze durations and products collected comparable to the baseline. In the visual high condition, the overall collection strategy was similar, but gaze was redirected away from the shelves (see also Table 3), suggesting that the visual distractors competed for attention without disrupting task execution. Yet, in the mental high condition, participants’ behaviors changed considerably: there were fewer grabs (reflecting lower performance, as reported in Table 3), more frequent revisits to the shopping list, and a longer overall proportion of time spent attending to the shopping list compared to the baseline (also observed in Table 3).

**Figure 7:**
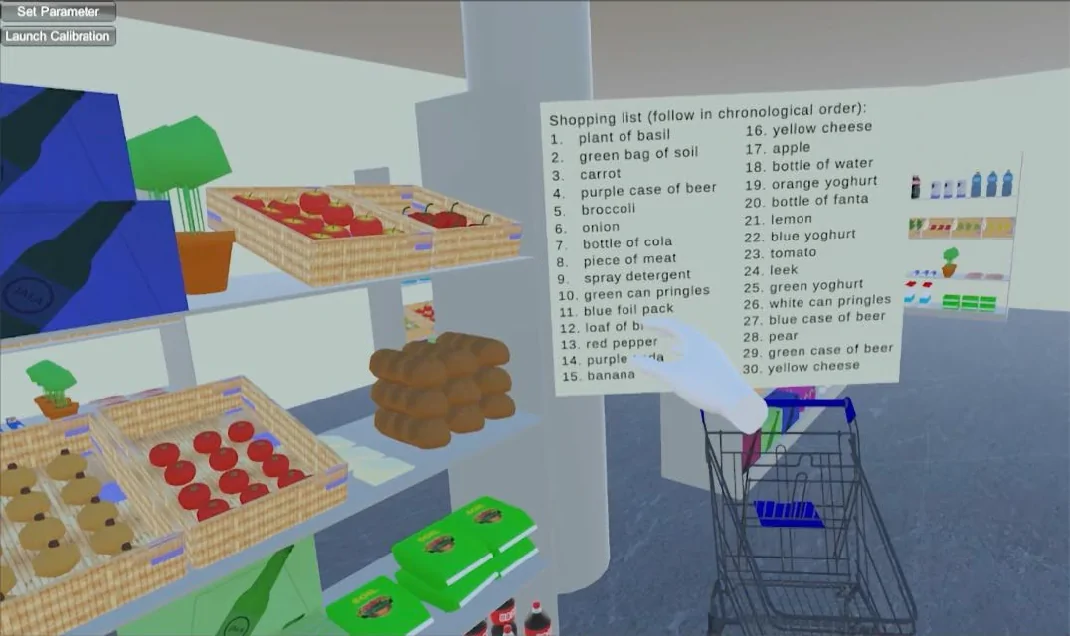
The immersive virtual supermarket from a first-person perspective during the mental high condition, showing an example of a finger-pointing strategy presumably used to identify the next product on the shopping list.

To further understand participants' behavior and strategies, we qualitatively analyzed video recordings showing their first-person perspective from the HMD during the shopping task. This analysis helped identify specific behaviors within the baseline and mental high conditions that could not be seen directly in the behavioral charts. For example, in the mental high condition, we observed that many participants adopted a step-by-step approach, prioritizing either the shopping or the 2-back task rather than performing both simultaneously; they would pause 1 task to execute the other. In 2 participants, we observed them using a finger-pointing strategy, presumably to assist with checking the shopping list (Figure 7), whereas in another case, 1 participant switched from English to Dutch (their native language) when performing the 2-back task.

## Discussion

### Principal Findings

This study examined how visual, auditory, and cognitive demands at 2 intensity levels influenced perceived workload, task performance, heart rate, gaze behavior, and head movement during a virtual grocery shopping task in immersive VR. The results are discussed below in relation to the 2 hypotheses.

Hypothesis 2, which predicted that cognitive demands would negatively affect task execution, was supported. Under mental high, the 2-back task substantially increased MDE, reduced task performance, prolonged head-stillness, and shifted gaze away from the shelves toward the shopping list. The 0-back condition showed a similar but attenuated pattern. This interference likely reflects competition for working memory, as both the n-back and the shopping task require maintaining and updating information in memory, creating a central bottleneck despite their different input and output modalities [15]. Heart rate was significantly elevated under mental low demand; the mental high effect did not reach corrected significance in the 2-tailed paired-samples *t* test, possibly because reduced physical activity under mental high partly offset any cognitive heart rate increase. Both mental conditions involved speaking aloud, which may also have contributed to the heart rate elevation relative to baseline.

Hypothesis 1, which predicted that high sensory demands would primarily distract attention and increase head movement, was only partially supported. Visual distractors redirected gaze toward other elements without reducing performance. Auditory demands increased self-reported MDE but had limited effects on behavior or performance. Neither modality increased head movement. Unlike the n-back task, these sensory manipulations were passive distractors that did not compete for working-memory resources, which likely explains their limited behavioral impact.

### Comparison With Prior Work

In the visual high condition, participants spent significantly less time gazing at the main shelves and significantly more time gazing at other elements compared to the baseline. A similar pattern was observed in the visual low condition, although there the decrease in gaze on the main shelves did not reach statistical significance after Bonferroni correction. These findings suggest that the nontask-relevant visual elements captured attention at the expense of task-relevant areas. However, this gaze redistribution did not affect performance or perceived workload. Contrary to the head-movement prediction in hypothesis 1, head-stillness duration did not differ from baseline under any visual or auditory condition. This pattern may be explained by perceptual load theory [39]: if the shopping task involved low perceptual load, spare capacity would allow processing of peripheral elements, explaining the gaze redistribution. The absence of a performance cost may reflect that the time spent glancing at distractors was short enough to be absorbed without reducing the total number of products placed in the cart. In the auditory high condition, participants reported significantly higher MDE than at baseline. The spatialized, semantically varied, and dynamically changing sounds from varying distances were likely difficult to ignore, imposing additional cognitive effort to sustain attention on the core task and thereby increasing perceived workload. Although task-irrelevant sounds and demanding acoustic conditions can affect performance in other settings [12,40], the auditory demands in this study did not significantly affect shopping performance, visual attention distribution, or head-stillness.

Mean heart rate was approximately 90 bpm across all 7 conditions, compared with approximately 105 bpm in [14], where participants were instructed to work as accurately and quickly as possible. The present instructions not to rush likely reduced the physical intensity of the task. Because heart rate reflects both physical [23] and mental demands [25], interpreting condition differences requires considering both components. Under mental high, participants collected significantly fewer products (~11 vs ~19 at baseline), meaning they probably moved less. This reduced physical activity may have suppressed heart rate, partly offsetting any increase associated with higher cognitive load and helping to explain why the raw mental high vs baseline difference was modest. An exploratory linear mixed-effect model adjusting for the total number of products placed in the cart suggested that a cognitive component may remain after accounting for reduced number of products collected (Table S3 and Figure S1 in Multimedia Appendix 1). However, the model estimates heart rate at product counts that rarely occurred within each condition and should be interpreted as an extrapolation-dependent sensitivity analysis. Speaking aloud in both mental conditions could also have contributed to their heart rate elevation relative to baseline, as speech has been shown to increase autonomic arousal [41]; the present data cannot fully separate this speaking component from a cognitive one. Converging evidence for a cognitive contribution comes from our earlier study [14], in which all participants collected the same number of products by design; heart rate was still significantly higher under cognitive demands (~108 vs ~102 bpm), supporting a cognitive effect that does not depend on the product-adjusted model.

The behavioral analysis revealed several adaptations to increased cognitive demands. Under mental high, participants tended to prioritize either the shopping or the 2-back task rather than performing both simultaneously, a pattern broadly consistent with the compensatory task-serialization strategies described by Boer et al [42] in older drivers at intersections. Participants in the mental high condition also frequently looked back at the shopping list and, in some cases, pointed toward it with their virtual hand (Figure 7). Despite collecting fewer products, participants spent substantially more time gazing at the shopping list than at baseline, suggesting that the increase in list-checking reflects cognitive load rather than simply being proportional to the number of products collected. This increased list-checking may reflect cognitive offloading, where individuals rely on external information to reduce demands on working memory [33,35]. The finger-pointing behavior observed in some participants may have served a comparable function, as pointing can support the processing of spatially separated information [34] and gestures have been shown to reduce working memory load [43,44] and improve comprehension [45], consistent with cognitive-load theory principles [46]. Gaze on other elements did not differ significantly between mental high and baseline. However, tracking loss duration did increase significantly under mental high. This suggests that high cognitive load may have increased spontaneous blinking, in line with research showing that blink rate may increase under higher cognitive load [47,48]. Yet, hardware tracking loss is an imperfect blink proxy, as gaps can also reflect headset slippage, extreme gaze angles, or downward gaze toward the cart. Only the 100-400 milliseconds range corresponds to canonical blink durations; the longer gaps observed under mental high are more susceptible to these alternative sources.

Prolonged gaze on AOIs may also reflect increased processing demands, consistent with research showing that higher cognitive load affects oculomotor behaviors such as fixation duration [27-29,49]. Head-stillness also increased significantly under high mental demand. However, because participants collected fewer products and therefore moved less in the mental high condition, this increase may partly reflect reduced physical task activity rather than a purely cognitive effect. Monitoring such behavioral adaptations may serve as a noninvasive method for estimating user state in IVR, as eye-tracking can provide behavioral information in IVR [50], and IVR behavioral metrics, including head and hand movement patterns, have been proposed as objective indicators of cognitive load [51]. Cucinella et al [14] previously noted that physiological measures exhibit large individual differences and require person-specific baselines to be practical. Combining performance metrics with observable behaviors such as gaze patterns and pointing gestures could offer a more accessible approach to workload assessment, potentially enabling IVR systems to adapt task complexity in real time to the user’s capacity.

### Limitations

This study has several limitations. First, the sample consisted of 22 unimpaired university students, and it is unclear whether the findings generalize to other populations, such as older adults or individuals with cognitive impairments. Older adults may also use compensatory strategies to support everyday functioning [52]. In contrast, cognitively impaired populations, for example, individuals with acquired brain injury, often show altered sensitivity to visual and auditory demands, ranging from hyposensitivity to hypersensitivity [53], and may rely less on compensatory behaviors, as awareness deficits following brain injury can limit recognition of one’s own impairments and the spontaneous use of compensatory strategies [52,54,55]. Accordingly, the present findings may underestimate the impact of sensory manipulations in clinical populations. Future studies should involve larger and more diverse samples, including participants of different ages, cognitive abilities, and cultural backgrounds.

Second, product-shelf assignments were fixed per condition rather than randomized per participant. The 4-shelf grouping constraint held fully only for the first 16 list positions in the experimental conditions (the first 28 positions in baseline), though most participants collected fewer than 19 products. We cannot fully exclude that differences in product placement across conditions influenced task difficulty. Third, participants’ search strategies (eg, scanning shelves left-to-right) were not quantified. Future studies should examine whether such strategies influence vulnerability to distractions. Fourth, while the increased gaze duration on the shopping list may reflect cognitive offloading [33], alternative explanations such as forgetting the target product, losing one’s place on the list, or pausing at the list during task switching cannot be excluded. Future studies could include task-related error metrics (eg, order of collection, missing products) to more directly assess memory strategies. Finally, although the n-back task is a widely used cognitive task [56], it may not fully reflect real-world shopping demands. Future studies could adopt realistic secondary tasks, such as answering a phone call or sending a text message [57], embedded within a naturalistic virtual shopping environment.

### Conclusions

The cognitive (2-back) task had a strong impact on perceived workload, performance, and behavioral measures, while visual and auditory demands primarily affected gaze distribution and perceived workload, respectively. Unlike prior work, this study compares 2 demand levels against a no-demand baseline and adds gaze and head-movement measures not previously examined. The absence of significant performance decrements under visual and auditory demands suggests that IVR environments can incorporate sensory richness without large reductions in task execution in this sample of unimpaired young adults, although the present design had limited power to detect small effects. Heart rate effects may have been partly masked by reduced physical activity under high cognitive load, limiting the use of heart rate as a standalone workload indicator in this task. By contrast, participants spontaneously adopted observable behavioral strategies to cope with high cognitive load, such as task serialization and increased list-checking. These behavioral adaptations, detectable from gaze and head movement data without physiological sensors, may offer a more practical approach to workload monitoring in IVR. As these findings were obtained with unimpaired young adults, their generalizability to clinical populations remains to be established. Future work in neurorehabilitation and training applications should investigate whether such behavioral markers can support the dynamic adjustment of task difficulty and environmental complexity to the individual user’s capacity.

## Data Availability

The anonymized data are publicly available in the Zenodo research data repository [58].

## Appendix

Multimedia Appendix 1 Linear mixed-effects model results.

## Abbreviations

AOI: area of interest
ECG: electrocardiogram
EDA: electrodermal activity
HMD: head-mounted display
IBI: interbeat interval
IVR: immersive virtual reality
MCAR: missing completely at random
MDE: mental demand and effort
TLX: task load index
