# Supplementary material for: Behavioral and Physiological Responses to Sensory and Cognitive Demands in an Immersive Virtual Reality Shopping Task in Unimpaired Young Adults: Within-Subjects Experimental Study
Source: JMIR Serious Games. 2026 Aug 28;14:e84893. doi: 10.2196/84893 (PMC13570242; doi:10.2196/84893)
Supplement: Supplementary file 1 [file games_v14i1e84893_app1.docx]

**Linear Mixed-Effects Model Results**

This appendix reports the results of linear mixed-effects models (LMEs) fitted to all dependent measures as a robustness check alongside the paired-samples *t* tests reported in Table 3. All 7 conditions were modeled simultaneously with a random intercept per participant, pooling residual variance across conditions. Two models (A, B) were fitted for each dependent measure. A third model (C), adding the total number of products placed in the cart as a covariate, was fitted for heart rate only as a sensitivity analysis to explore whether the observed heart rate pattern might partly reflect differences in motor activity across conditions.

**Models**

***Model A:*** *DV ~ Condition + (1 | Participant)*. This model estimates the effect of each condition relative to Baseline (reference category) with a random intercept per participant. No covariates.

**Model B:** *DV ~ Condition + Order + (1 | Participant)*. This model adds presentation order (trial position 1-7) as a linear covariate to control for learning or fatigue effects across the experimental session.

**Model C:** *DV ~ Condition + Order + Products + (1 | Participant).* This model adds the total number of products placed in the cart as a second covariate. Model C was fitted for heart rate only. Because participants collected substantially fewer products under Mental high (~11) than under Baseline (~19), the model effectively estimates heart rate at product counts that rarely occurred within each condition. Model C is therefore presented as a sensitivity analysis. Figure S1 visualizes the degree of extrapolation for heart rate.

**Definitions**

**Condition:** Categorical factor with 7 levels (Baseline, Visual low, Visual high, Auditory low, Auditory high, Mental low, Mental high). Baseline is the reference category in all models.

**Order:** The position (1-7) in which the condition was presented to each participant. Included as a linear fixed effect to account for learning or fatigue.

**Products:** The total number of products placed in the cart in a given trial. Included as a linear fixed effect to control for differential motor activity.

**(1 | Participant):** Random intercept per participant, absorbing between-subject variability (eg, some participants generally have higher heart rates or report higher workload).

**β (beta):** Fixed-effect coefficient for the condition, representing the estimated mean difference from Baseline in the original units of the dependent variable.

**SE:** Standard error of β.

***t*:** *t*-statistic for the test of β=0 (residual degrees of freedom).

***P*:** *P* value for the *t* test.

**σ_u_:** Standard deviation of the random intercept, reflecting between-subject variability in overall level.

**σ_e_:** Residual standard deviation, reflecting within-subject variability after accounting for fixed effects. Because the random intercept absorbs between-subject differences, σ_e_ is analogous to the standard deviation of paired difference scores.

***d***_z,A_**, *d***_z,B_**, *d***_z,C_**:** Within-subject effect sizes from Models A, B, and C, respectively. Computed as β / (σ_e_ × √2) for each model, following the convention used in the emmeans R package (Lenth, 2024) for paired contrasts in mixed models. The denominator σ_e_ × √2 corresponds to the expected standard deviation of paired difference scores under compound symmetry, making *d*_z,A_, *d*_z,B_, and *d*_z,C_ directly comparable to the classical Cohen *d*_z_ values reported in Table 3. The pooled residual σ_e_ is estimated across all 7 conditions simultaneously, whereas the classical *d*_z_ in Table 3 uses the pair-specific standard deviation of difference scores. Consequently, the LME-based values may differ from the classical values.

Comparing *d*_z,A_, *d*_z,B_, and *d*_z,C_ reveals how the effect size changes when controlling for order and motor activity: *d*_z,A_ is unadjusted (analogous to *d*_z_ from the main text); *d*_z,B_ is adjusted for presentation order; *d*_z,C_ is adjusted for both order and total number of products placed in the cart. Because Model C was fitted for heart rate only, the other tables report only *d*_z,A_ and *d*_z,B_.

Bonferroni-corrected significance threshold: *P*<.008 (.05/6).

**Table S1.** Self-reported mental demand and effort (MDE) (0 to 100)

*Model A:* σ_u_=10.34, σ_e_=11.70. *Model B:* σ_u_=10.41, σ_e_=11.26.

|  | Mean | *SD* | *d*_z,A_ | *d*_z,B_ |
| --- | --- | --- | --- | --- |
| Baseline | 22.05 | 16.56 |  |  |
| Visual low | 21.59 | 14.71 | −0.03 | 0.01 |
| Visual high | 27.61 | 14.87 | 0.34 | 0.38 |
| Auditory low | 27.84 | 15.95 | 0.35 | 0.51 |
| Auditory high | 39.09 | 20.17 | 1.03 | 1.05 |
| Mental low | 41.93 | 15.41 | 1.20 | 1.26 |
| Mental high | 74.77 | 13.34 | 3.19 | 3.34 |

**Table S2.** Performance (total number of products placed in the cart)

*Model A:* σ_u_=2.57, σ_e_=2.67. *Model B:* σ_u_=2.66, σ_e_=2.01.

|  | Mean | *SD* | *d*_z,A_ | *d*_z,B_ |
| --- | --- | --- | --- | --- |
| Baseline | 18.68 | 2.70 |  |  |
| Visual low | 19.73 | 4.86 | 0.28 | 0.23 |
| Visual high | 18.55 | 3.66 | −0.04 | −0.16 |
| Auditory low | 19.18 | 3.17 | 0.13 | −0.28 |
| Auditory high | 17.50 | 4.24 | −0.31 | −0.35 |
| Mental low | 18.23 | 4.23 | −0.12 | −0.20 |
| Mental high | 10.68 | 3.26 | −2.12 | −2.89 |

**Table S3.** Mean heart rate (bpm)

*Model A:* σ_u_=13.49, σ_e_=3.38. *Model B:* σ_u_=13.49, σ_e_=3.35. *Model C:* σ_u_=12.35, σ_e_=3.09.

|  | Mean | *SD* | *d*_z,A_ | *d*_z,B_ | *d*_z,C_ | β_C_ | *SE*_C_ | *t*_C_ | *P* value_C_ |
| --- | --- | --- | --- | --- | --- | --- | --- | --- | --- |
| Baseline | 87.46 | 12.78 |  |  |  |  |  |  |  |
| Visual low | 87.33 | 14.71 | −0.02 | −0.03 | −0.12 | −0.52 | 0.95 | −0.55 | .59 |
| Visual high | 86.40 | 14.66 | −0.21 | −0.23 | −0.18 | −0.78 | 0.95 | −0.82 | .41 |
| Auditory low | 86.49 | 13.26 | −0.11 | −0.18 | −0.07 | −0.32 | 0.97 | −0.33 | .74 |
| Auditory high | 87.97 | 15.95 | 0.11 | 0.12 | 0.28 | 1.24 | 0.94 | 1.32 | .19 |
| Mental low | 91.47 | 15.26 | 0.84 | 0.84 | 1.00 | 4.38 | 0.94 | 4.67 | <.001 |
| Mental high | 90.28 | 13.53 | 0.59 | 0.58 | 1.93 | 8.42 | 1.44 | 5.85 | <.001 |

**Table S4.** Head-stillness duration (s)

*Model A:* σ_u_=5.53, σ_e_=4.33. *Model B:* σ_u_=5.57, σ_e_=4.00.

|  | Mean | *SD* | *d*_z,A_ | *d*_z,B_ |
| --- | --- | --- | --- | --- |
| Baseline | 14.89 | 5.82 |  |  |
| Visual low | 15.26 | 6.14 | 0.06 | 0.13 |
| Visual high | 14.71 | 5.93 | −0.03 | 0.02 |
| Auditory low | 14.98 | 6.17 | 0.01 | 0.23 |
| Auditory high | 16.13 | 8.11 | 0.20 | 0.19 |
| Mental low | 15.72 | 5.96 | 0.14 | 0.17 |
| Mental high | 28.44 | 10.77 | 2.21 | 2.43 |

**Table S5.** Gaze duration on the shopping list (s)

*Model A:* σ_u_=4.04, σ_e_=4.37. *Model B:* σ_u_=4.06, σ_e_=4.23.

|  | Mean | *SD* | *d*_z,A_ | *d*_z,B_ |
| --- | --- | --- | --- | --- |
| Baseline | 31.21 | 6.10 |  |  |
| Visual low | 31.70 | 5.67 | 0.08 | 0.04 |
| Visual high | 31.43 | 3.93 | 0.04 | 0.01 |
| Auditory low | 33.59 | 4.66 | 0.39 | 0.26 |
| Auditory high | 32.25 | 5.71 | 0.17 | 0.19 |
| Mental low | 36.17 | 6.82 | 0.80 | 0.82 |
| Mental high | 41.10 | 8.60 | 1.60 | 1.63 |

**Table S6.** Gaze duration on the main shelves (s)

*Model A:* σ_u_=4.26, σ_e_=5.12. *Model B:* σ_u_=4.28, σ_e_=5.00.

|  | Mean | *SD* | *d*_z,A_ | *d*_z,B_ |
| --- | --- | --- | --- | --- |
| Baseline | 73.53 | 6.67 |  |  |
| Visual low | 70.70 | 5.54 | −0.39 | −0.37 |
| Visual high | 67.42 | 5.38 | −0.84 | −0.84 |
| Auditory low | 70.90 | 6.38 | −0.36 | −0.26 |
| Auditory high | 72.77 | 5.17 | −0.11 | −0.13 |
| Mental low | 69.74 | 6.79 | −0.52 | −0.53 |
| Mental high | 60.29 | 10.38 | −1.83 | −1.85 |

**Table S7.** Gaze duration on the cart (s)

*Model A:* σ_u_=2.22, σ_e_=1.86. *Model B:* σ_u_=2.23, σ_e_=1.83.

|  | Mean | *SD* | *d*_z,A_ | *d*_z,B_ |
| --- | --- | --- | --- | --- |
| Baseline | 8.91 | 2.50 |  |  |
| Visual low | 9.01 | 3.57 | 0.04 | 0.06 |
| Visual high | 8.18 | 3.03 | −0.28 | −0.26 |
| Auditory low | 8.15 | 2.84 | −0.29 | −0.20 |
| Auditory high | 8.50 | 3.20 | −0.16 | −0.17 |
| Mental low | 7.43 | 2.66 | −0.57 | −0.57 |
| Mental high | 7.62 | 2.82 | −0.49 | −0.48 |

**Table S8.** Gaze duration on other elements (s)

*Model A:* σ_u_=0.00, σ_e_=2.60. *Model B:* σ_u_=0.00, σ_e_=2.60.

|  | Mean | *SD* | *d*_z,A_ | *d*_z,B_ |
| --- | --- | --- | --- | --- |
| Baseline | 2.92 | 0.98 |  |  |
| Visual low | 5.21 | 2.19 | 0.62 | 0.63 |
| Visual high | 9.70 | 4.35 | 1.84 | 1.86 |
| Auditory low | 3.51 | 3.19 | 0.16 | 0.20 |
| Auditory high | 3.13 | 1.29 | 0.06 | 0.05 |
| Mental low | 2.91 | 1.64 | −0.00 | −0.00 |
| Mental high | 3.98 | 3.23 | 0.29 | 0.30 |

**Table S9.** Summary of covariate effects across all dependent measures

| Measure | Order  β (Model B) | Order  *P* value (Model B) |
| --- | --- | --- |
| MDE | −1.524 | .001 |
| Performance | 0.843 | <.001 |
| Heart rate | 0.228 | .11 |
| Head-stillness | −0.796 | <.001 |
| Gaze list | 0.514 | .004 |
| Gaze shelves | −0.536 | .01 |
| Gaze cart | −0.159 | .04 |
| Gaze other | −0.096 | .37 |

The Order coefficient indicates the change in the dependent variable per position in the presentation sequence (1-7), controlling for condition. For heart rate, the Products coefficient indicates the change per additional product collected, controlling for condition and order. Mental high vs Mental low contrast (Model C): diff=4.05 bpm, 95% CI 1.31 to 6.78, *F*(1,142) = 8.56, P=.004. In Model C, the Products coefficient was β=0.689 (P<.001).

**Figure S1.** Per-participant mean heart rate as a function of the total number of products placed in the cart for the Baseline (gray circles) and Mental high (red circles) conditions. Gray lines connect the same participant across conditions. Condition means are shown as a gray square (Baseline) and a red diamond (Mental high). The dotted gray line and dash-dot red line show the LME Model C predictions for Baseline and Mental high, respectively, evaluated at the mean presentation order; their common slope is the Products coefficient (β=0.689 bpm per product) and their constant vertical separation is the Model C condition effect (β=8.42 bpm). The dark red bracket illustrates this separation at the Mental high mean product count (~11). Because Baseline participants collected approximately 19 products on average, the Baseline prediction line extends well beyond the observed Baseline data in the low product-count region, making the degree of extrapolation visually apparent.
